# Peanut Oral Immunotherapy in Children: An Italian Single-Center Retrospective Cohort Study

**DOI:** 10.3390/nu18081252

**Published:** 2026-04-16

**Authors:** Benedetta Pessina, Camilla Sertori, Simona Barni, Francesco Catamerò, Giulia Liccioli, Erika Paladini, Lucrezia Sarti, Leonardo Tomei, Julia Upton, Claudia Valleriani, Francesca Mori, Mattia Giovannini

**Affiliations:** 1 Allergy Unit, Meyer Children’s Hospital IRCCS, 50139 Florence, Italy; 2 Department of Health Sciences, University of Florence, 50139 Florence, Italy; 3 Clinical Pediatrics, Department of Molecular Medicine and Development, University of Siena, 53100 Siena, Italy; 4 Division of Immunology and Allergy, Department of Paediatrics, The Hospital for Sick Children, Toronto, ON M5G 1E8, Canada; 5 Temerty Faculty of Medicine, University of Toronto, Toronto, ON M5S 1A8, Canada; 6 Immunology Laboratory, Meyer Children’s Hospital IRCCS, 50139 Florence, Italy

**Keywords:** children, food allergy, IgE, peanut, oral immunotherapy, anaphylaxis

## Abstract

**Introduction:** Peanut oral immunotherapy (P-OIT) is an emerging treatment strategy for peanut allergy (PA). Although a standardized pharmaceutical product, Peanut (*Arachis hypogaea*) Allergen Powder-dnfp, has been approved in several countries, it is not universally available. In such contexts, real-world protocols using readily utilizable peanut products may represent an alternative approach. This study aimed to describe the feasibility, safety, and clinical outcomes of P-OIT using toasted peanuts in a real-world effort in a pediatric population. **Methods:** This single-center retrospective cohort study enrolled children who initiated P-OIT at our tertiary pediatric hospital Allergy Unit between April 2015 and December 2024. Demographic and clinical features, allergy test results, and information about P-OIT were recorded. Desensitization was defined as tolerance of 630 mg of peanut protein (PP). **Results:** Sixty patients (51.7% male; median age 8.2 years) were included. 22/60 (36.7%) achieved desensitization within a median time of 22.7 months. 21/60 (35%) were still undergoing P-OIT at a median tolerated dose of 100 mg of PP, and 17/60 (28.3%) discontinued treatment, most commonly due to loss to follow-up (44%). At least one adverse reaction occurred in 43/60 (71.7%) patients, predominantly mild and self-limiting (68.3% resolved spontaneously, 39.5% occurred at home). However, 11/60 (18.3%) showed anaphylaxis, and 3/60 (5%) received adrenaline. A reduction in Ara h 2 serum-specific IgE levels compared to the baseline was observed in patients completing escalation (*p* = 0.03). **Conclusions:** In this real-world single-center cohort, P-OIT using toasted peanuts was feasible in a subset of patients and was associated predominantly with mild adverse reactions, although systemic reactions were also recorded. Treatment discontinuation and adherence remain relevant challenges. These findings highlight the need for prospective, controlled studies to better define the role, safety profile, and patient selection criteria for food-based P-OIT protocols in settings where standardized products are not available.

## 1. Introduction

Peanut allergy (PA) is one of the most common and potentially severe food allergies in children, with a prevalence of approximately 1–2% in Western countries, as confirmed by oral food challenge (OFC) studies [1,2,3]. Data from the European Anaphylaxis Registry indicate that peanuts are a leading cause of anaphylactic reactions in the pediatric population, responsible for 26.3% of anaphylaxis cases in children and 18.3% in adolescents [4]. Unlike other common food allergies, such as milk, egg, and soy allergies, which often resolve during early childhood or adolescence, PA tends to persist if untreated, with approximately 75–80% of affected individuals continuing to experience hypersensitivity reactions in adulthood [1].

The diagnosis of PA is typically supported by a combination of clinical history, serum-specific IgE (s-IgE) testing, skin tests, and, when necessary, OFC [5]. Component-resolved diagnosis is now fully part of the diagnostic flowchart, and it may even support the stratification of the risk of anaphylaxis during OFC [6,7]. The four major components associated with severe allergic reactions are Ara h 1, Ara h 2, Ara h 3, and Ara h 6, with Ara h 2 and Ara h 6 being the best predictors of severe systemic reactions to peanuts that require adrenaline treatment [8].

Currently, the standard of care for PA involves strict dietary avoidance and the use of emergency medications, such as adrenaline auto-injectors, in case of accidental exposure [9]. The chronic nature of PA places a considerable burden on both patients and their families, restricting food choices, complicating social engagement, and contributing to reduced quality of life, as well as increased consumption of peanuts in our diets, increasing anxiety related to food consumption [10,11].

In recent years, oral immunotherapy (OIT) has emerged as a promising treatment strategy aimed at decreasing the reactivity to food by means of incremental oral administration of food protein to induce desensitization, raising the reactivity threshold to a certain food allergen [12,13].

Peanut (*Arachis hypogaea*) Allergen Powder-dnfp (PTAH) or AR101, commercially known as Palforzia, is the only standardized oral drug approved by the US Food and Drug Administration (FDA) and the European Medicines Agency (EMA) for use in children aged 1 to 17 years with confirmed PA [14,15,16].

PTAH is the first standardized OIT product approved for PA, providing a rigorously validated treatment protocol that ensures consistent dosing and comprehensive monitoring, thus overcoming the variability and limitations of earlier experimental OIT regimens. Despite its potential, PTAH is not yet widely available in all clinical settings, and several questions remain regarding optimal patient selection, long-term outcomes, and real-world safety and efficacy [17,18]. Further research is needed to better understand which children are most likely to benefit from peanut OIT (P-OIT) with PTAH and how to tailor treatment protocols accordingly. Moreover, its health costs may be considerable, and regular consumption of peanuts may not be possible even after PTAH treatment.

In countries where PTAH is not readily available, OIT using toasted peanut as a food product remains a feasible treatment option. This product is readily available for purchase in all Italian supermarkets, and its consumption in the general population is widespread owing to its common use as a snack. Other studies using peanut commercial products (such as peanut flour, boiled peanuts, peanut butter, and peanut-coated corn puffs) showed promising results in terms of efficacy but significant rates of adverse reactions [19,20,21,22,23]. In fact, using a non-standardized product may cause variability in terms of the milligrams (mg) of protein in the prepared doses, protocol employment, and rate of adverse reactions [24,25,26]. However, the most reported adverse reactions are local, such as itching of the oropharynx and perioral rashes.

The primary objective of this study was to assess the feasibility of P-OIT by using toasted peanuts in a real-world Italian pediatric population with PA. The secondary objectives were to assess its safety and efficacy and to describe the clinical and allergy characteristics of an Italian pediatric cohort with PA.

## 2. Methods

### 2.1. Study Design

This single-center retrospective cohort study enrolled children undergoing P-OIT at the Allergy Unit of Meyer Children’s Hospital IRCCS, Florence, Italy, from April 2015 to December 2024. The inclusion criteria were (1) a confirmed diagnosis of IgE-mediated PA and (2) consent to undergo P-OIT. The diagnosis of PA was confirmed using the following criteria: (1) history of anaphylaxis to peanut and positive prick-by-prick (PbP) and/or s-IgE to peanut or, in the absence of history of anaphylaxis, (2) positive peanut open, unblinded OFC following the protocol described by Barni et al. [27]. Exclusion criteria were any contraindications to undergoing OIT according to the current guidelines [28].

Demographic and clinical features, allergy test results, co-allergies, comorbidities, and information regarding P-OIT were recorded.

Electronic medical charts were reviewed, and data such as sex (female/male), age (in months), family history of atopy (present/absent), presence or absence of atopic comorbidities (asthma, allergic oculorhinitis, atopic dermatitis, and other food allergies), and history of reactions to peanuts (clinical signs and symptoms, treatment) were collected. To minimize selection and recall bias, we applied uniform eligibility criteria across all participants and relied on electronic medical charts compiled simultaneously by nurses and physicians.

Skin prick tests (SPTs) for the main pollen allergens (grass pollen, pellitory, mugwort, cypress, olive tree, hazel tree, birch pollen, and poplar tree) and PbP tests for peanut were performed and considered positive if the wheal diameter was ≥3 mm at the 15-min reading. Histamine (10 mg/mL; Lofarma, Milan, Italy) and normal saline were used as the positive and negative controls, respectively. PbP tests are performed by piercing fresh peanuts with a sterile lancet and then immediately applying the lancet to the patient’s skin, following standard allergy testing procedures. Peanut s-IgE and s-IgE to Ara h 1, Ara h 2, Ara h 3, Ara h 8, and Ara h 9 were determined using a commercial assay (ImmunoCAP system, Thermo Fisher Scientific, Uppsala, Sweden) with a positive cut-off point of 0.1 kU/L.

### 2.2. P-OIT Protocol

According to the standard practice in our Allergy Unit, P-OIT was proposed for all patients with the abovementioned criteria and was initiated in the Day Hospital regimen in the Allergy Unit of our hospital.

In summary, an initial dose of 5 mg of toasted peanut (1.3 mg of peanut protein, PP) was administered and then increased every 30 min according to the following scheme: 5 + 10 + 25 + 50 + 100 + 150 + 300 + 600 + 1200 mg of toasted peanut if the patient showed no reactions. If any objective skin, respiratory, cardiovascular, neurologic, or gastrointestinal manifestations were observed, patients were promptly treated and advised to continue at home with the last tolerated dose at the OFC. The P-OIT protocol consists of an induction period, with subsequent hospital visits every 3–6 months to increase the dose. Between visits, patients were instructed to take the dose at least 3 times/week after a meal at home, to avoid physical activity in the hour before and 3 h after peanut ingestion, and to halve the dose in case of acute intercurrent disease. Patients able to tolerate the final OFC step of 1200 mg of peanuts, with a cumulative challenge dose of 2440 mg (roughly five peanuts, corresponding to 630 mg of PP), were considered desensitized. The primary efficacy endpoint was the achievement of clinical desensitization (defined as tolerance of 630 mg of PP). Other outcomes, such as sustained unresponsiveness (SU), were not assessed. Patients who successfully reached a final single OIT dose of 1200 mg were not allowed to consume peanuts ad libitum. Instead, they were instructed to continue ingesting this specific maintenance dose at least three times per week under controlled conditions (e.g., after meals, avoiding exercise before and after ingestion). Patients who permanently discontinued treatment or were lost to follow-up were classified as non-desensitized for the primary endpoint.

Data on adverse reactions, such as any clinical manifestation occurring during the build-up or maintenance phase, whether at home or in the clinic, were recorded, including the type of reaction, presence of cofactors, and utilized therapy.

Informed consent/assent was obtained from parents/legal guardians and children when appropriate for all procedures. This study was approved by the Ethic Committee Tuscany Region—Pediatric (Reference #195/2025, 14 October 2025).

### 2.3. Statistical Analysis

Statistical analyses were performed using IBM Statistical Package for Social Science software (SPSS, Version 28.0, Chicago, IL, USA). No formal power analysis was performed because the study included all eligible patients within the predefined timeframe, making the sample size determined by data availability rather than by prospective calculation. Non-normally distributed variables were presented as medians, while categorical variables were presented as percentages. Continuous variables were compared using the nonparametric Wilcoxon rank-sum test, whereas categorical variables were compared using the chi-squared test. To identify the factors associated with OIT success, we performed a logistic regression analysis using the occurrence of reactions during OIT and the finalization of OIT as outcome (dependent) variables. Univariate analyses identified associated independent variables for the multivariate model fitted using a backward elimination procedure (likelihood ratio was used as a criterion, and *p* > 0.2 for removal). The Hosmer-Lemeshow test was used to evaluate the goodness of fit of the multivariable model. The results are expressed as odds ratios (ORs) and 95% confidence intervals (CIs). All statistical tests were two-sided, and statistical significance was set at *p* < 0.05.

## 3. Results

### 3.1. P-OIT Population

Sixty patients who underwent P-OIT were enrolled in the study. Of these, 31/60 (51.7%) were male, with a median age at first reaction of 36.6 months (IQR 24–77.2) and a median age at the beginning of P-OIT of 99.1 months (IQR 60.6–144.8). The vast majority of patients (52/60, 86.7%) were ≥4 years old at the beginning of P-OIT induction.

**Figure 1:**
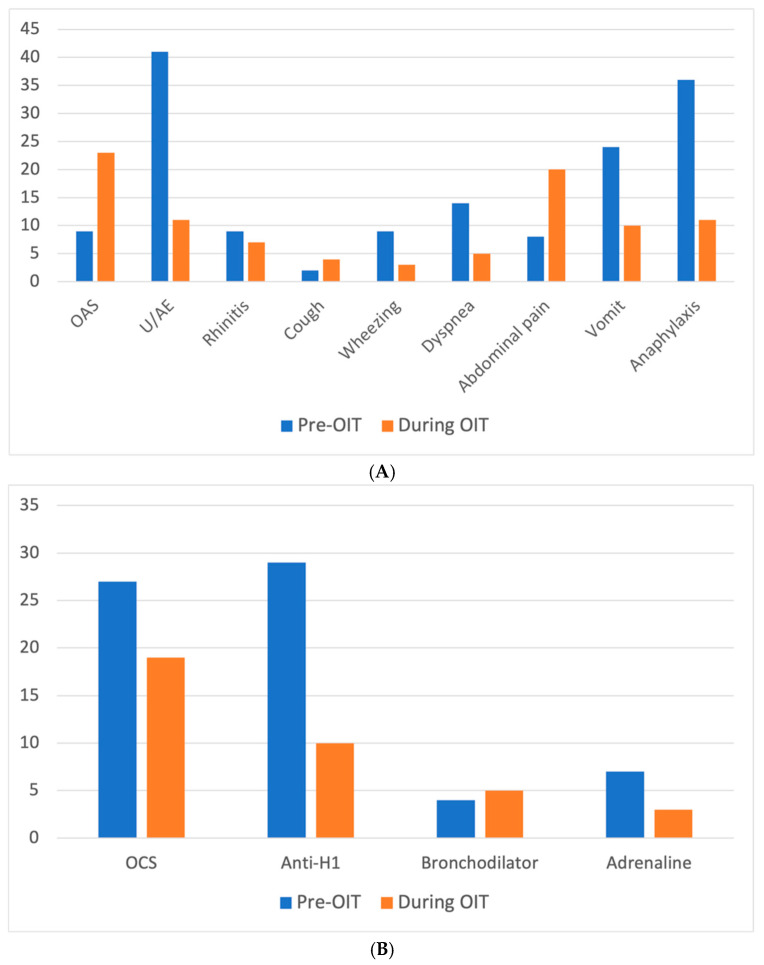
(**A**) Clinical signs and symptoms frequency upon first allergic reaction to peanut and reactions during OIT. (**B**) Adopted treatment for first allergic reaction to peanut and for reactions during OIT. Anti-H1: antihistamine; OAS: oral allergy syndrome; OCS: oral corticosteroid; OIT: oral immunotherapy; U/AE: urticaria/angioedema.

A family history of atopy was present in 39/60 (65%) patients, and atopic comorbidities were common: atopic dermatitis in 18/60 (30%), allergic oculorhinitis in 18/60 (30%), and asthma in 13/60 (21.7%). One patient (1/60, 1.2%) showed well-controlled eosinophilic esophagitis (EoE). Demographic characteristics are shown in Table 1. The rate of co-sensitization to tree nuts was high, with 26/60 (43.3%) showing co-sensitization to at least one tree nut. Pollen sensitization was also present in many patients, especially for grass pollen (34/58, 58.6%), olive tree pollen (25/58, 43.1%), and birch pollen (17/58, 29.3%). For 2/60 patients, information about pollen sensitization was missing. A clinical history of anaphylaxis to peanuts was reported in 36/60 children (60%), while the most common reaction at onset was urticaria/angioedema (41/60, 68.3%) (Figure 1A). Treatment for the first reaction was mainly oral corticosteroids (27/52, 51.9%), oral antihistamines (29/52, 55.8%), or inhaled bronchodilators (4/52, 7.7%), while only 8/60 (15.4%) patients with anaphylaxis were treated with intramuscular adrenaline (Figure 1B). For 8/60 patients, information regarding the treatment of the first reaction was not available in the medical chart. All 24 patients without a history of anaphylaxis showed a reaction at open low-dose OFC, with a median reaction dose of 100 mg of peanuts (IQR 2.5–450). Baseline allergy tests are presented in Table 2, showing a strong sensitization to the peanut storage protein Ara h 2.

### 3.2. P-OIT Feasibility

P-OIT was initiated with a median latency of 37.7 months (IQR 18.9–90.8) since the first reported reaction. Of the 60 patients enrolled for P-OIT, 22/60 (36.7%) were desensitized with a median duration of 22.7 months (IQR 10.8–28.7), 21/60 (35%) were still undergoing P-OIT induction with a median tolerated dose of 400 mg of peanut (100 mg of PP), while 17/60 (28.3%) abandoned P-OIT and were therefore classified as non-desensitized for the primary endpoint.

Among those who abandoned P-OIT, 11/17 (64.7%) were lost to follow-up, while for the others the primary reasons for discontinuing P-OIT were adverse reactions (5/17, 29.4%), taste aversion (2/17, 11.7%), fear of reaction (2/17, 11.7%), family reasons (1/17, 5.8%), and exacerbation of pre-existing EoE (1/17, 5.8%).

In patients who achieved desensitization, allergy tests were repeated at the end of the treatment, and a significant reduction was observed in Ara h 2 s-IgE levels (10.6 kU/L, IQR 1.33–42.75, at the start vs. 0.93 kU/L, IQR 0.31–7.94, at the end, *p* = 0.03) (Table 2). No significant changes were observed in Ara h 1, Ara h 3, Ara h 8, and Ara h 9 s-IgE levels before and after P-OIT.

### 3.3. P-OIT Safety

Among patients who started P-OIT, 43/60 (71.7%) showed at least one adverse reaction, of which 17/43 (39.5%) were at home. All reactions occurring at home were self-reported by the patients’ families.

Reactions were mainly non-systemic and self-limiting, with 23/60 (38.3%) being oral manifestations (Figure 1A). Other common adverse reactions included abdominal pain (20/60, 33.3%), urticaria (11/60, 18.3%), and vomiting (10/60, 16.7%), while the less common signs and symptoms were rhinitis (7/60, 11.7%), dyspnea (5/60, 8.3%), wheezing (4/60, 6.7%), and cough (3/60, 5%). Overall, 19/60 (31.7%) patients required medical treatment, whereas the rest resolved spontaneously.

Nonetheless, 11/60 (18.3%) patients experienced anaphylaxis, five of which occurred at home; all other anaphylactic episodes occurred at dose increases in the hospital under medical supervision. Three patients with anaphylaxis were treated with intramuscular adrenaline (3/60, 5%), two of these at home, whereas the others were managed with oral corticosteroids and/or antihistamine therapy (Figure 1B). Among the 11/60 patients who showed systemic reactions, four interrupted treatment. Two of them received adrenaline, but only one declared “reactions” as the reason to interrupt OIT. Instead, five of them achieved desensitization, and two are still undergoing treatment, of which one received adrenaline.

Cofactors were identified in four adverse reactions (4/60, 6.7%), of which one was anaphylaxis. In one case, the cofactor was not specified; in two other cases, the reaction was concomitant with an infection, and in one case with alcohol consumption.

### 3.4. Baseline Characteristics Associated with Desensitization

**Figure 2:**
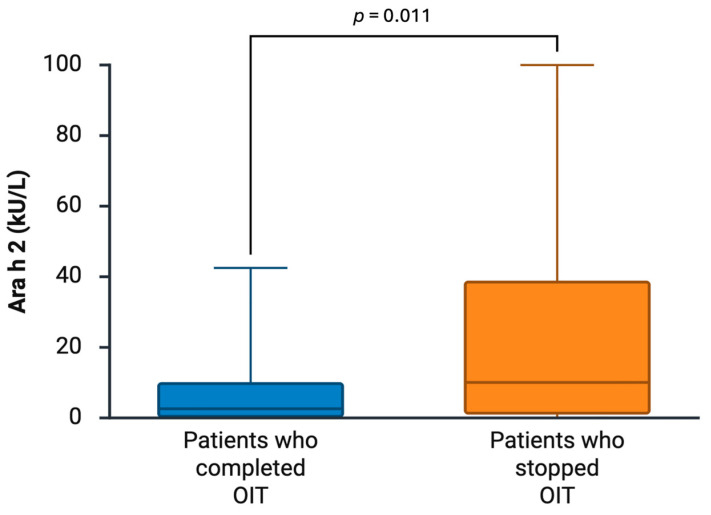
Difference between baseline Ara h 2 s-IgE levels (in kU/L) in patients who stopped (N = 17/60) versus patients who completed OIT induction (N = 22/60). OIT: oral immunotherapy. Created with BioRender.com.

In the univariate analysis, unsuccessful desensitization was associated, to the limit of significance, with atopic dermatitis (*p* = 0.06) and higher baseline peanut s-IgE levels (*p* = 0.075). Median Ara h 2 s-IgE levels were significantly different between patients who completed compared to patients who stopped OIT (2.76 kU/L, IQR 0.43–13.7, versus 22.65 kU/L, IQR 4.79–52.22, *p* = 0.011) (Figure 2). In the multivariate analysis, no variable was associated with the outcome of desensitization (Table 3), although this analysis was performed with an exploratory aim.

## 4. Discussion

Our study is the first real-world study to retrospectively explore P-OIT using commercial materials (toasted peanuts) in a pediatric Italian population.

Our results seem to suggest that P-OIT with toasted peanuts in this setting is feasible; however, further studies are needed to confirm its safety and efficacy. Our findings align with a growing body of evidence supporting the potential of P-OIT for managing PA, including the use of PTAH. However, our study aimed to provide insights into the practical application of P-OIT using readily available peanuts, which may be particularly relevant in settings where PTAH is not accessible.

The rationale for using commercial toasted peanuts is primarily that these are commonly consumed in the Italian diet; therefore, they are extremely easy to access. Moreover, the specific properties of the food matrix are likely to influence both allergen structure and absorption kinetics, but we did not analyze this aspect in our study. For example, it is known that the roasting process induces high thermal denaturation and the formation of protein aggregates, reducing the susceptibility of major allergens (Ara h 1 and Ara h 2) to gastric pepsin digestion and increasing peanut allergenicity [29]. Toasting is generally performed at lower temperatures and for shorter durations than roasting and, therefore, might have a less pronounced effect on allergenicity. The high lipid content of the peanut matrix plays a crucial role in gastrointestinal physiology, significantly delaying gastric emptying, as shown by Mackie et al. [30]. This results in a slower, more controlled delivery of the allergen to the intestinal mucosa. All these factors likely modulate the absorption profile of P-OIT and should be investigated for toasted peanuts in appropriately designed studies, which ideally compare the different matrices.

In our cohort, approximately 40% of the patients were able to achieve clinical desensitization to roughly 630 mg of PP. Our desensitization rate includes patients who discontinued therapy or were lost to follow-up, thus providing a conservative estimate of real-world effectiveness. In previous randomized controlled studies, the efficacy in terms of desensitization rate was reported to be between 60 and 70%, as proven at exit double-blind, placebo-controlled, food challenge (DBPCFC) [31]. Unfortunately, direct comparisons between studies are often difficult to make because of different definitions of desensitization [32].

In the STOP II phase 2 randomized controlled trial (RCT), after 6 months of maintenance therapy with 800 mg of PP, 24/39 children (62%) passed the challenge with 1400 mg of PP (versus no patients in the placebo group) [33].

Moreover, in the first trials exploring the efficacy of AR101, it was shown that a lower maintenance dose could provide similar results. In the PALISADE trial, after gradual up-dosing from 0.5 to 300 mg of PP, 23/29 (79%) and 18/29 (62%) children passed the OFC with 443 mg and 1043 mg of PP, respectively. Among the 26 patients who received a placebo, only 5/26 (19%) and 0/26 (0%) had passed the same challenges [34]. In phase III of the same trial, after 12 months of AR101 therapy, 250/372 participants (67.2%) passed a challenge with 600 mg of PP versus 5/124 participants (4%) who received a placebo [35]. Another noteworthy study, the POSEIDON study, confirmed the same outcomes in younger children (1–4 years): 73.5% of PTAH-treated participants tolerated 600 mg of PP at exit DBPCFC compared with only 6.3% in the placebo group [36].

Subsequent trials have explored the possibility of SU after a period of avoidance. In a recent randomized DBPCFC by Jones et al. [37], after 2.5 years of AR101 therapy, tolerance was achieved in 68/96 (71%) children compared to 1/50 (2%) placebo-treated subjects. After a period of avoidance, remission rates were still in favor of the treatment group, even if with lower rates compared to desensitization: 20/96 (21%) treated subjects still retained peanut tolerance compared to 1/50 (2%) placebo-treated children. This was confirmed in a subsequent study on patients aged <4 years, showing a potentially greater benefit for this younger population compared to older children. In fact, a trend was observed with higher remission rates in children younger than 24 months (71%) compared to 24–35.9 months (35%) and 36–47.8 months (19%).

In our study, exit OFC was not performed, and the overall desensitization rate was lower than what was reported in the literature. However, a proportion of patients were still undergoing OIT, and the efficacy may therefore be underestimated. Moreover, our data reflect real-world scenarios and include roughly a 1/3 of patients who interrupted OIT, mainly due to loss to follow-up, which was a common event in our cohort, especially in patients with high Ara h 2 s-IgE levels, highlighting the need for patient education and accurate patient selection before starting OIT. Nonetheless, almost 40% of our patients regularly tolerated five peanuts, and 35% were on regular consumption of 100 mg of PP, which may protect them from severe reactions upon accidental exposure [38]. Moreover, most patients in our cohort were >4 years when P-OIT was started, possibly due to delays in referral. Therefore, despite providing interesting data about the real-world compliance and feasibility of P-OIT in children, our study has scarce information about the cohort that potentially benefits the most from OIT, which is children aged <4 years.

The few other published real-world studies included a small number of patients and were consistent with our observations. An interesting analysis of 30 young (<36 months) patients undergoing P-OIT with commercial products showed a higher compliance compared to our cohort, with only 10% interruption of OIT, once again highlighting the possibility that an early start may be a winning solution, both to increase effectiveness and compliance, as well as to decrease the risk of severe reactions [39]. In the first P-OIT study performed solely on an adult population of 21 patients aged 19–39 years, six patients dropped out of the study (28.5%), but 67% met the primary endpoint of tolerating at least 1400 mg of PP on intention-to-treat analysis [40]. These data suggest that P-OIT with real-world materials may be more difficult to perform with increasing age; however, this is an effective treatment option, contrary to what was previously suggested by the PALISADE trial, with only 42% of adult participants passing the exit DBPCFC versus 14% of placebo-receiving participants.

When assessing safety, a considerable number of patients experienced reactions during dose escalation. In our study, reactions were recorded per patient rather than per administered dose, and thus dose-level reaction rates were not available due to the retrospective design. Nevertheless, most patients showed only OAS or abdominal pain. In other studies, gastrointestinal side effects of P-OIT have been reported to be the most common [33,35,41]. Systemic reactions are rarer and reported as 2–5% in more controlled studies [36]. In our cohort, anaphylaxis occurred in roughly 1/5 of the patients, with 5% requiring adrenaline treatment. This rate is comparable to that of another real-world study, the DEVIL trial, which reported approximately a 2/3 rate of reactions during up-dosing, with a 4% rate of adrenaline administration [41]. The same rate (4%) was reported in the SmaChO RCT [22], where 2/50 children were treated with adrenaline but 5/50 showed severe reactions, and in the studies by Soller and Grzeskowiak [19,20]. Our study highlights the underutilization of adrenaline, which is now a well-established gap in the management of food allergy [42].

In an adult study by Hunter et al., most adverse reactions were grade 1 (95.4% of adverse reactions), while nine patients showed grade 2 reactions and one patient showed a grade 4 reaction [40]. Anaphylactic episodes that occurred outside the hospital were exclusively associated with a known cofactor (running/infection) or previous missed doses, and three patients self-administered a single dose of epinephrine on four separate occasions. This reflects the importance of continuous patient education and strict follow-up at all ages. In our case, this was obviated by being reachable every day from the family and guiding the patients through any dose modifications upon reaction. However, in the pediatric age group, cofactor identification may be more difficult, and the family must be adequately warned about cofactor avoidance.

It is apparent by now that the main difficulty for the clinician is still navigating between different existing P-OIT protocols [42,43]. In this regard, studies such as the DEVIL study compared the efficiency and safety of low-dose (300 mg of PP) and high-dose (3000 mg of PP) OIT and reported that the desensitization (85% vs. 75%) and SU rate (85% vs. 71%) did not differ significantly between the low-dose and high-dose groups [41]. In our study, the maintenance dose was an intermediate dose (600 mg of PP), and the up-dose was usually doubled, both of which may be modulated to reduce adverse reactions. It is hypothesized that maintaining a lower dose could be a strategy to increase compliance, especially in patients with high Ara h 2 s-IgE levels.

A secondary result of our study is the evidence of a reduction in Ara h 2 s-IgE levels between the pre- and post-induction periods, which may be a marker of successful desensitization, as described in previous studies [44]. Other molecular components were not significantly changed in our cohort, possibly because of insufficient sample size. However, due to the design of our study, the reduction in Ara h 2 s-IgE cannot be considered a surrogate marker of clinical efficacy. Nonetheless, better and more complete molecular definition of the patient in different studies may help in predicting the potential for OIT success or SU gain. A second major challenge for clinicians dealing with OIT is to identify potential risk factors for OIT interruption or loss of desensitization. The high rate of loss at follow-up is a notable challenge in current practice, and further studies should explore strategies to improve adherence in real-world P-OIT programs.

This real-world study highlights all these gaps but shows potential effectiveness and partial feasibility of P-OIT with commercial products. The sample size and the retrospective design of our study limit the significance of some of our findings, and certainly, more studies are needed to develop and validate predictive models that could support and guide personalized OIT strategies. In previously published studies, lower peanut-induced basophil activation and lower peanut s-IgE levels were associated with better treatment outcomes and lower risk of reactions [45,46,47].

Our study has several limitations. Its small sample size and retrospective design render it susceptible to selection and recall biases. A long referral time may be the cause of a delay between the first reaction and OIT start, which may impact its outcomes. Its single-center design without a control group makes it mainly descriptive and hypothesis-generating, preventing causal inferences.

Because of the real-world setting, no OFC was performed at the end of escalation, and it did not address the longer effects of OIT, such as whether desensitization can be maintained without daily peanut consumption following several years of continuous OIT, to achieve SU.

Moreover, because the study was conducted in a tertiary center, these results may not be generalizable to less specialized settings. Future trials are required to confirm our preliminary findings.

Finally, this study may stimulate discussion about P-OIT safety and efficacy and its impact on pediatric patients’ quality of life and that of their caregivers [48].

## 5. Conclusions

In this single-center real-world pediatric cohort, P-OIT using toasted peanuts was implementable in a tertiary Italian care setting and allowed a proportion of patients to achieve clinical desensitization to 630 mg of PP. This dietary protocol offers a possible alternative to pharmaceutical products where these are not available, potentially improving patients’ quality of life due to their widespread availability. However, treatment discontinuation and adherence highlight important practical challenges in routine care. Adverse reactions were common but predominantly mild and manageable; nevertheless, systemic reactions, including anaphylaxis, occurred and require careful supervision, structured patient education, and strict follow-up. While food-based P-OIT may represent a pragmatic alternative in settings where standardized pharmaceutical products are not available, future prospective multicenter studies with large cohorts and long follow-up periods are needed to validate these results and optimize personalized protocols for peanut allergy management.

## Figures and Tables

**Table 1:** Demographic and clinical characteristics of our cohort.

Characteristic	Total N	N (%)	Median	IQR
Age at reaction (months)	60		36.6	24–77.2
Sex, female	60	29 (48.3%)		
Family history of atopy	60	39 (65%)		
Atopic dermatitis	60	18 (30%)		
Allergic rhinitis	60	18 (30%)		
Asthma	60	13 (21.7%)		
Age at P-OIT (months)	60		99.1	60.6–144.8
Pollen co-sensitization				
Grass pollen	58	34 (58.6%)		
Olive tree	58	25 (43.1%)		
Birch pollen	58	17 (29.3%)		
Co-sensitization to tree nuts	60	26 (43.3%)		

**Table 2:** Median total IgE and specific IgE for peanut with molecular components at baseline and at the end of P-OIT for all patients who completed P-OIT escalation (N = 22/60). Statistical significance was set at *p* < 0.05 and statistically significant results are reported in bold.

kU/L	Pre-OIT		Post-OIT		
	Median	IQR (Q1–Q3)	Median	IQR (Q1–Q3)	*p* Value
Total IgE	383	140–709	501	299–1047	1.00
s-IgE peanut	12.90	4.15–55.50	2.83	1.79–16.00	0.60
s-IgE Ara h 1	0.95	0.02–11.80	0.08	0.02–0.70	0.59
s-IgE Ara h 2	**10.06**	**1.33–42.75**	**0.93**	**0.31–7.94**	**0.03**
s-IgE Ara h 3	0.07	0.01–1.99	0.03	0.02–0.04	0.73
s-IgE Ara h 8	0.02	0.00–0.28	0.98	0.02–4.29	0.06
s-IgE Ara h 9	0.00	0.00–0.09	0.03	0.01–4.75	0.48

**Table 3:** Baseline variables associated with unsuccessful desensitization in univariate and multivariate analysis. CI = confidence intervals; OIT = oral immunotherapy; PbP = prick-by-prick test; s-IgE = serum-specific IgE.

	Odds Ratio (95% CI)	
Variable	Univariate Analysis	Multivariate Analysis
Age at beginning	1.000 (0.988–1.013)	
Female sex	1.350 (0.379–4.804)	
Family history of atopy	0.667 (0.178–2.491)	
Atopic dermatitis	4.000 (0.945–16.925) *p* = 0.060	1.949 (0.385–9.854) *p* = 0.420
Asthma	0.453 (0.076–2.690)	
Allergic rhinitis	0.538 (0.130–2.223)	
Co-allergy with tree nuts	1.969 (0.542–7.145)	
Baseline PbP wheal diameter	1.082 (0.875–1.339)	
Peanut s-IgE	1.022 (0.998–1.046) *p* = 0.075	0.981 (0.957–1.006) *p* = 0.138
History of anaphylaxis	0.816 (0.223–2.992)	
Latency from reaction to OIT (time in months)	0.999 (0.995–1.003)	

## Data Availability

The raw data supporting the conclusions of this article will be made available by the authors on request.
